# Implementation and Evaluation of a Local CBT Teaching Programme for Core Psychiatry Trainees

**DOI:** 10.1192/bjo.2024.283

**Published:** 2024-08-01

**Authors:** James Bloomfield, Eimear Leyden, Alina Vaida

**Affiliations:** Coventry and Warwickshire Partnership NHS Trust, Warwick, United Kingdom

## Abstract

**Aims:**

Core Psychiatry Trainees (CTs) are required to complete two psychotherapy cases, utilising different therapeutic modalities as part of their training. During supervision sessions, CTs in Coventry and Warwickshire reported feeling underprepared to start their psychotherapy cases. Locally, the most frequently used modality for short cases is Cognitive Behavioural Therapy (CBT). Here we evaluate a local CBT teaching program implemented to prepare CTs, delivered by the trust Psychotherapy Tutor in conjunction with a CT, Dr Bloomfield, who has experience delivering CBT in a talking therapies service.

**Methods:**

We implemented a teaching programme which consisted of 30-minute teaching sessions occurring immediately after Balint groups, which are usually well attended. Dr Bloomfield designed a teaching plan, with separate CBT teaching topics divided into 12 sessions. Each session focused on a CBT concept with practical examples. The effectiveness of psychotherapy teaching was evaluated with pre- and post-teaching online surveys assessing preparedness, confidence in formulation, and knowledge of specific techniques. The survey consisted of Likert scales ranging from 1–10 with lower numbers indicating a negative response. Trainees were also surveyed about Psychodynamic Psychotherapy in the absence of specific teaching, as a comparator.

**Results:**

13 CTs responded to the pre-teaching survey and 16 CTs to the post teaching survey, with near-equal weighting across the training grades. Responses indicated a median increase across all areas for CBT, with trainees reporting higher confidence in formulation (7 to 8.5), improved knowledge of CBT techniques (5 to 8) and increased preparedness to start seeing patients (5 to 7). By comparison, there was a modest increase in preparedness (5 to 6) and confidence in psychodynamic formulation (3 to 3.5), with knowledge of specific aspects of Psychodynamic Psychotherapy unchanged (6 and 6).

**Conclusion:**

Regional teaching alone may not be sufficient in preparing trainees to start seeing psychotherapy patients. The CBT learning needs of CTs in Coventry and Warwickshire were effectively met by providing a series of short, tailored sessions covering the different aspects of CBT, resulting in improved confidence, preparedness, and knowledge of CBT techniques. The same increases were not seen in trainees’ responses regarding Psychodynamic Psychotherapy, though small increases were seen in preparedness and confidence in formulation. Our next phase of the teaching will focus on Psychodynamic Psychotherapy teaching, with further repetition of the survey.

